# Immunological priming of mesenchymal stromal/stem cells and their extracellular vesicles augments their therapeutic benefits in experimental graft-versus-host disease *via* engagement of PD-1 ligands

**DOI:** 10.3389/fimmu.2023.1078551

**Published:** 2023-02-16

**Authors:** Alexander Hackel, Sebastian Vollmer, Kirsten Bruderek, Stephan Lang, Sven Brandau

**Affiliations:** Department of Otorhinolaryngology, University Hospital Essen, University Duisburg-Essen, Essen, Germany

**Keywords:** mesenchymal stromal/stem cells (MSCs), graft-versus-host disease (GVHD), immunomodulation, extracellular vesicles (EVs), regulatory T cells (Tregs), cytokine priming, programmed death ligand system (PD-1 and PD-L1)

## Abstract

Mesenchymal stromal cells (MSCs) and their extracellular vesicles (EVs) exert profound anti-inflammatory and regenerative effects in inflammation and tissue damage, which makes them an attractive tool for cellular therapies. In this study we have assessed the inducible immunoregulatory properties of MSCs and their EVs upon stimulation with different combinations of cytokines. First, we found that MSCs primed with IFN-γ, TNF-α and IL-1β, upregulate the expression of PD-1 ligands, as crucial mediators of their immunomodulatory activity. Further, primed MSCs and MSC-EVs, compared to unstimulated MSCs and MSC-EVs, had increased immunosuppressive effects on activated T cells and mediated an enhanced induction of regulatory T cells, in a PD-1 dependent manner. Importantly, EVs derived from primed MSCs reduced the clinical score and prolonged the survival of mice in a model of graft-versus-host disease. These effects could be reversed *in vitro* and *in vivo* by adding neutralizing antibodies directed against PD-L1 and PD-L2 to both, MSCs and their EVs. In conclusion, our data reveal a priming strategy that potentiates the immunoregulatory function of MSCs and their EVs. This concept also provides new opportunities to improve the clinical applicability and efficiency of cellular or EV-based therapeutic MSC products.

## Graphical Abstract

Multi-cytokine priming combined with subsequent screening of immune responding MSCs, improved MSC-EVs immunomodulatory activity in a PD-1 dependent manner. Primed MSC-EVs demonstrated immunosuppressive effects on activated T cells and mediated an enhanced induction of regulatory T cells. Importantly, primed MSC-EVs reduced disease severity and prolonged survival in a mouse model of acute graftversus-host disease (GvHD). These effects could be reversed by adding neutralizing antibodies directed against PD-Ls to MSC-EVs.

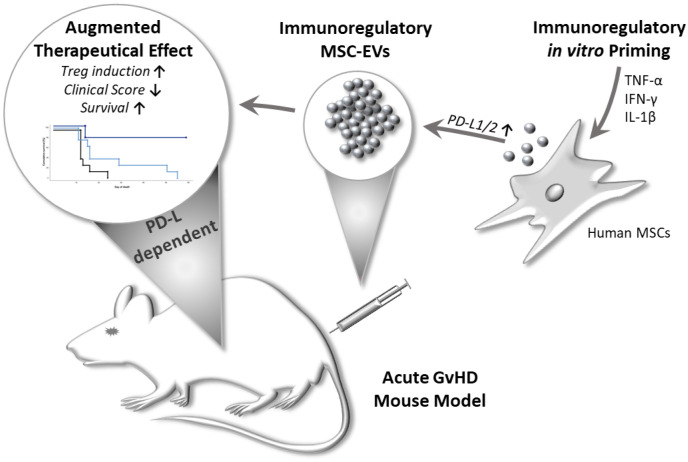

## Introduction

Mesenchymal stromal/stem cells (MSCs) are non-hematopoietic, fibroblast-like progenitor cells, capable of differentiating into different mesenchymal tissue lineages, such as chondrocytes, osteoblasts and adipocytes (1–4). They are defined by their plastic adherence, the expression of a set of characteristic cell surface markers, but absence of endothelial and hematopoietic cell surface antigens, and their multilineage differentiation capacity (1, 5–7). Originally identified in the bone marrow, they have now been isolated from many vascularized tissue sources and body fluids (8–13). Considering their broad immunoregulatory capacity towards the innate and adaptive immune system, MSCs are in the focus as a novel therapeutic approach in many inflammation-related diseases (14–17).

MSCs display their multifaceted immunomodulatory properties *via* both, cell-contact-dependent direct mechanisms and contact-independent paracrine mechanisms, including the induction of anti-inflammatory dendritic cells (DCs) and Tregs (18–22). While initial studies have indicated that MSCs are capable of migrating to areas of tissue damage, more recent studies suggest that MSCs often do not reach these sites, but rather accumulate in the lung and spleen and are rapidly cleared from the system (11–13, 23–25). Thus, immunomodulation exerted by MSCs is strongly associated with paracrine mechanisms, e.g. extracellular vesicles (EVs) are suggested as potential mediators of their therapeutic effects (26–28).

EVs contain a multitude of bio- and immuno-active molecules, such as cytokines, nucleic acids, and other proteins, which in part resemble a comparable molecular spectrum to their parental cells of origin (29, 30). Regarding their immunoregulatory activity, MSC-derived EVs exert comparable therapeutic effects akin to the MSCs themselves (26, 31, 32). Compared to their parental cells and conventional MSC-based therapy, the use of MSC-EVs represent a more easy-to-handle sterile therapeutic tool, whose application also minimizes any risks for patients (11–13, 26, 33).

The receptor programmed cell death 1 (PD-1) system is a crucial component in the regulation and activation of T cells, as demonstrated by the enhanced susceptibility of PD-1 knockout mice to autoimmune diseases (34, 35) and its role in GvHD mice models (36–38). The expression of PD-1 ligand 1 (PD-L1) is reported on non-hematopoietic cells, like MSCs, but also on hematopoietic cells, while PD-1 ligand 2 (PD-L2)-expression is typically found on antigen-presenting cells (APCs), but it is also found to be expressed by MSCs (37, 39, 40). Previous reports have suggested the presence of PD-L1 within EVs and as soluble ‘free’ entities, and in addition as part of soluble cell membrane particles (37, 41, 42).

A big challenge in MSC and EV therapy is to overcome the considerable variations in therapeutic efficiency observed between different donor and manufacturing batches (43). Variations in culture conditions, differences in donor and tissue origin, but also variations in isolation and culture procedures can alter the epigenetic profile of MSCs, thus providing a challenge to generate immunoregulatory MSCs/EVs with consistent properties (44). MSC biology itself may provide some important cues to generate higher degree of reproducibility. Indeed, exposure to an inflammatory environment is necessary to fully activate MSCs immunoregulatory function to a more robust level of homogeneity (45, 46).

In our previous studies, assessing the immune response of MSCs to stimulations with multiple cytokines (47), we have identified an optimal proinflammatory cytokine stimulation approach for MSCs to be employed prior to their therapeutic application, for generating fully activated MSCs and MSC-EVs with an increased and robust immunoregulatory capacity. Building on our previous studies (46, 48), we here used tissue-specific MSCs derived from two different sources, nasal mucosa and human bone marrow, to evaluate their immunomodulatory features and the underlying mechanism in an *in vivo* mouse model.

Ultimately, this method may provide a more efficient and robust therapeutic approach to better standardize MSC/EV-based therapy of inflammation-related diseases.

## Methods

### Study approval, isolation and culture of MSCs

The use of human samples was approved by the ethics committee of the medical faculty of the University Duisburg-Essen. Nasal mucosa MSCs, further referred to as “MSCs” in this study, were obtained from the inferior nasal concha of healthy individuals (age 30-70 years) at the Department of Othorhinolaryngology, University Hospital Essen (Essen, Germany). The isolation, culture of MSCs and evaluation of differentiation potential were conducted as described before (8). MSCs were cultured in DMEM/RPMI-1640 high glucose (50%/50% v/v), supplemented with 2mM L-Glutamine, 100 IU/ml penicillin, 100 mg/ml streptomycin (all ThermoFisher Scientific, Karlsruhe, Germany) and 10% (v/v) heat-inactivated FCS (Merck/Biochrom, Berlin, Germany). All MSCs used in experiments were between passages 3-6.

Bone marrow MSCs further referred to as “bmMSCs” were kindly provided by Bernd Giebel from the Institute of Transfusion Medicine, University Hospital Essen, Germany, registered as “MSC 41.5”. BmMSCs were originally isolated from bone marrow aspirates of healthy individuals after informed consent as described before (26) and acquisition was approved by the ethics committee of the medical faculty of the University Duisburg-Essen. Phenotyping of bmMSCs used in the study was conducted in line with ISCT minimal criteria for MSCs (6), by evaluating cell-surface marker expression with flow cytometry and trilineage differentiation to validate multipotent differentiation capacity of MSCs (8). Experiments with bmMSCS were conducted within passage 4-6. BmMSCs were cultured in DMEM low glucose (PAN Biotech, Aidenbach, Germany), supplemented with 10% platelet lysate (kindly provided by the Institute of Transfusion Medicine, University Hospital Essen), 100 U/mL penicillin-streptomycin-glutamine and 5 IU/mL Heparin (Ratiopharm, Ulm, Germany).

### Multi cytokine-priming of MSCs and bmMSCs

Cytokine-priming of MSCs and bmMSCs was based on a previously established concept (47). In brief, MSCs and bmMSCs were stimulated in culture medium, with IFN-γ (1000 U/ml; PeproTech, Hamburg, Germany) and TNF-α (1000 U/ml; Miltenyi, Bergisch Gladbach, Germany) in the present or absence of IL-1β (10 ng/ml; Miltenyi) for 24 h at 37°C, 5% CO_2_. Afterwards, cells were washed twice with PBS, and incubated in culture medium for additional 48 h. Subsequently, MSCs were either processed directly for FACS analysis, co-culture experiments or administered in mouse GvHD models.

### Flow cytometric analysis

Following antibodies were used for MSC characterization: CD29-PE (clone MAR4), CD45-V500 (clone HI30, both BD Bioscience, Heidelberg, Germany), CD31-APC-eFlour780 (clone WM59), CD73-PerCP-eFlour710 (clone AD2, both ThermoFisher scientific), CD34-FITC (clone 581), CD90-Brilliant Violet 421 (clone 5E10) and CD105-Pe-Cy7 (clone 43A3, all BioLegend, Koblenz, Germany). After stimulation with IFN-γ/TNF-α +/- IL-1β cells were stained with CD54-APC (clone HA58), CD274-PerCP-eFlour710 (PD-L1, clone MIH1, all ThermoFisher scientific) and CD273-PE-Vio770 (PD-L2, clone MIH18, Miltenyi). Cells were analysed using FACSCanto II flow cytometer and BD FACS Diva Software 8.0. (BD Bioscience).

### Isolation and size characterization of extracellular vesicles from MSCs and bmMSCs

For isolation of MSCs and bmMSCs EVs, cells were cultured and stimulated with IFN-γ/TNF-α +/- IL-1β as described above in Nunc™ High Cell Factory™. Cell culture supernatants were collected and EVs were purified by differential centrifugations and polyethylene glycol (PEG) precipitation as recently described (49). EV isolated from culture medium of 4*10^7^ MSCs or bmMSCs that had been conditioned for 48 h were defined as 1 EV unit. MSC-EV size and particle concentration were determined by using nanoparticle tracking analysis by ZetaView (Particle Metrix, Meerbusch, Germany) (49, 50). ZetaView was calibrated with a polystyrene bead standard of 100 nm (ThermoFisher Scientific). Loaded samples were recorded by video at 11 positions, repeated 5 times. Further settings were Sensitivity: 75, shutter: 75, minimum brightness: 20, minimum size: 5, maximum size: 20 and median value: 20.

### Transmission electron microscopy of extracellular vesicles

Transmission electron microscopy of extracellular vesicles was executed in the department of Physical Chemistry, Faculty of Chemistry, University Duisburg-Essen, Essen, Germany. The MSC-EV preparations were diluted 1:10 (1 EV-unit/ml in 10 mM HEPES, 0.9% NaCl) and subjected to a formvar-coated copper grid. The samples were further incubated with a staining solution of 0.75% Uranyl formate, 6 mM NaOH and dried at room temperature. MSC-EV samples were analysed with a ZEISS EM910 at 120 kV.

### SDS-PAGE and Western blot analysis

For SDS-PAGE, supernatants and corresponding EV preparations were incubated with SDS sample buffer (pH 6.8, 50 mM Tris, 4% glycerine, 0.8% SDS, 0.04% bromphenol blue and with or without 1.6% β-mercaptoethanol) as described before (49). Samples were further analysed by wet immunoblotting on nitrocellulose membranes (GE Healthcare) and staining with following antibodies: mouse anti-human CD9 (VJ1, kindly provided by Francisco Sanchez-Madrid), mouse anti-human CD81 (JS-81, BD-Bioscience), rabbit anti-human/mouse/rat HSP70/HSPA1A (R&D Systems, Abingdon, United Kingdom), rabbit anti-human Flotillin-1 (Sigma-Aldrich, St. Loius, USA) and rabbit anti-human CD274 (PD-L1, Pro-Sci-Inc., Poway, USA). Goat anti-rabbit IgG and goat anti-mouse IgG (both HRP-conjugated, Cell Signaling Technology, Danvers, MA, USA) were used as secondary antibodies.

### CD3^+^ T cell proliferation assay

CD3^+^ T cells of healthy donors were isolated from peripheral blood mononuclear cells after density-gradient centrifugation *via* positive selection using human CD3^+^ MicroBeads (Miltenyi) according to the manufacturer’s instructions. After isolation, T cells were labelled with 10 mmol/l Cell Proliferation Dye eFluor 450 (CPDye405) according to the manufacturer’s instructions (ThermoFisher Scientifc). To assess the effect of MSC cells on CD3^+^ T cells, cells were co-cultured in MSC culture medium (see above) with a T-cell:MSC ratio of 2:1 (0.5*10^5^ CD3^+^: 0.25*10^5^ MSCs) at 37°C, 5% CO^2^. To study the influence of EVs from stimulated MSCs on T cell proliferation, 0,5*10^5^ CD3^+^ T cells were cultured in the present or absence of 30 µL isolated EV preparations. T cell proliferation was induced by adding tetrameric antibody-complex ImmunoCult™ Human CD2/CD3/CD28 (StemCell Technologies, Grenoble, France). CPDye405 intensity was analysed by flow cytometry after 4 days of proliferation. Proliferation index calculation is based on dye dilution and was calculated with ModFit LT 3.3 (Verity Software House) according to an algorithm provided by the software. The index of the non-proliferated fraction was subtracted, and the index of T cells without MSCs was set as 100%. The proliferation index is the sum of the cells in all generations divided by the computed number of original parent cells theoretically present at the start of the experiment. The proliferation index thus reflects the increase in cell number in the culture over the course of the experiment.

### CD3^+^ Tregs induction assay

After isolation by CD3 microbeads (see above), CD3^+^ T cells were stimulated with MSC or bmMSC preparations, in the respective culture medium (see above). Treg assay was performed in a 96-well round-bottom plate coated with antibodies against CD3 (10 µg/ml, clone OKT-3; ThermoFisher scientific) and CD28 (2 µg/ml, clone 28.2; Beckman Coulter) for T cell-activation. To assess the effect of MSC cells on CD3^+^ T cells, cells were co-cultured in a T-cell:MSC ratio of 2:1 (0.5*10^5^ CD3^+^: 0.25*10^5^ MSCs) at 37°C, 5% CO^2^ To test effects of EVs isolated from stimulated MSC, 0,5*10^5^ CD3^+^ T cells were cultured in the present or absence of 30 µl EV preparations. After 3 days of culture CD3^+^ T cells were stained with CD4-APC-Cy7 (clone RPA-T4), CD25-APC (clone NM-A251, both BD-Bioscience), CD127-PE-Cy7 (eBioRDR5, ThermoFisher scientific), and intracellular with FoxP3-FITC (ECH101, both ThermoFisher scientific). Tregs induction were determined with marker expression of CD4^+^ CD127^dim^ CD25^+^ FOXP3^+^ of total CD4^+^. Cells were analysed with BD FACSCanto II using BD FACS DIVA 8.01 software (BD Biosciences).

### GvHD mouse model

Female BalbALB/c and C57BL/6 mice (12-14 weeks old) were purchased from Charles River Laboratory or Janvier Laboratory, were housed in a pathogen-free facility of the University Hospital Essen and treated with water containing antibiotics (0,11 g/l Neomycin, Ampicillin, Vancomycin and Metronidazole). All animal procedures were performed in accordance with the international guidelines for good laboratory practice and the institutional guidelines of the University Hospital Essen, approved by the animal welfare committees of North Rhine Westphalia. MHC-mismatched murine HSCT model of GvHD was generated by transplanting CD90.2 depleted bone marrow cells (bm cells) from female C57BL/6 donor-mice into female Balb/c recipient-mice, previously total body irradiated with a dose of 8 Gy (50, 51). The recipient female BALB/c mice were reconstituted with 5*10^6^ bm cells from C57BL/6 mice and 0.5*10^6^ naïve CD4^+^ spleen cells were used to induce GvHD pathology. For CD90.2 depletion of total bone marrow cells after isolation from femur and tibia of C57BL/6 mice, negative selection mouse CD90.2 cell isolation Kit (Miltenyi, Bergisch Gladbach, Germany) according to the manufacturer’s instructions was used. Naïve CD4^+^ spleen cells were isolated from total spleen after erythrocyte depletion with ammonium-chloride-potassium buffer and subsequent negative selection using mouse naïve CD4^+^ T cell isolation Kit (Miltenyi) according to the manufacturer’s instructions. The clinical symptoms of GvHD were assessed with a clinical scoring system (**Supplementary Table S1**). In long-term experiments, mice were kept until day 58 after HSCT to analyse long-term clinical follow up and to record survival curves. In short-term experiment, mice were sacrificed on day 11 after HSCT and the frequency of Tregs in the circulation was determined. Treatment was performed by intravenous injection of 0.03 EV units per mice for three consecutive days or by a single treatment with 5*10^6^ MSC per mice, starting as soon mice regained weight at day 7 or 8 after BMT. Mice were sacrificed when the respective criteria as set out in the institutional and governmental animal welfare guidelines were reached (**Supplement Table**). For neutralizing PD-1 ligands in EV preparations, MSC-EVs were pre-incubated for 30 minutes with inhibitory antibodies against CD274 (PD-L1, clone 29E.2A3, BioLegend) (2ug/ml) and CD273 (PD-L2, MIH18, BioLegend) (2µg/ml). To determine non-specific effects of inhibitory antibodies, isotype controls were used at the same concentrations as the specific antibodies. Unbound antibodies were removed by 100-kDa molecular weight cut-off (Vivaspin^®^, Sartorius, Göttingen, Germany) centrifugal polyether sulfone membrane ultrafiltration before intravenous injection of EV preparations. Mice were sacrificed when the respective criteria as set out in the institutional and governmental animal welfare guidelines were reached. Animals that died from radiation disease or due to failed engraftment of bone marrow of C57BL/6 donor mice were excluded from experiments. Mice that had to be sacrificed during the experiment due to clinical scoring were continuously recorded with a score of 10.

### Swiss roll colon analysis

In order to analyse the colon histology of groups within the short-term GvHD model, we employed a previously published technique referred as “Swiss roll” (52). In brief, directly after sacrifice of mice feces were removed by flushing with PBS. The colon was rolled up on a wooden stick to be subsequently fixed in 4% formalin. The fixed preparations were embedded in paraffin for subsequent cutting in 5 μm sections by microtome. Sections were stained with hematoxylin and eosin (HE) and analysed by light microscopy.

### Analysis of blood samples

Blood samples were taken from donor C57BL/6 mice at day 1 and from recipient Balb/c mice on day 11 after irradiation. Mice were anaesthetized with isoflurane, blood was drawn from retro-orbital venous and collected in EDTA-tubes and subjected to flow cytometry analysis. Notably, it was not feasible to collect a sufficient quantity of blood samples from every mouse for further analysis, caused by the severe pathology of the GvHD mouse model.

### Statistical analysis

All data are shown as means as center value and errors bars (+/-) SD or SEM as indicated. Data were analysed by paired parametric t-test or by one-way analysis of variance (ANOVA) with Tukey’s multiple comparison test. Kaplan–Meier curves were analysed with Gehan-Breslow-Wilcoxon to compare survival between treatment groups. Data are presented as p-values of p < 0.05 (*), p < 0.01 (**), p < 0.001 (***) or p < 0.0001 (****) were considered statistically significant.

## Results

### Enhanced induction of Tregs by primed MSC and their EVs is mediated by PD-1 Ligands *in vitro*

**Figure 1:**
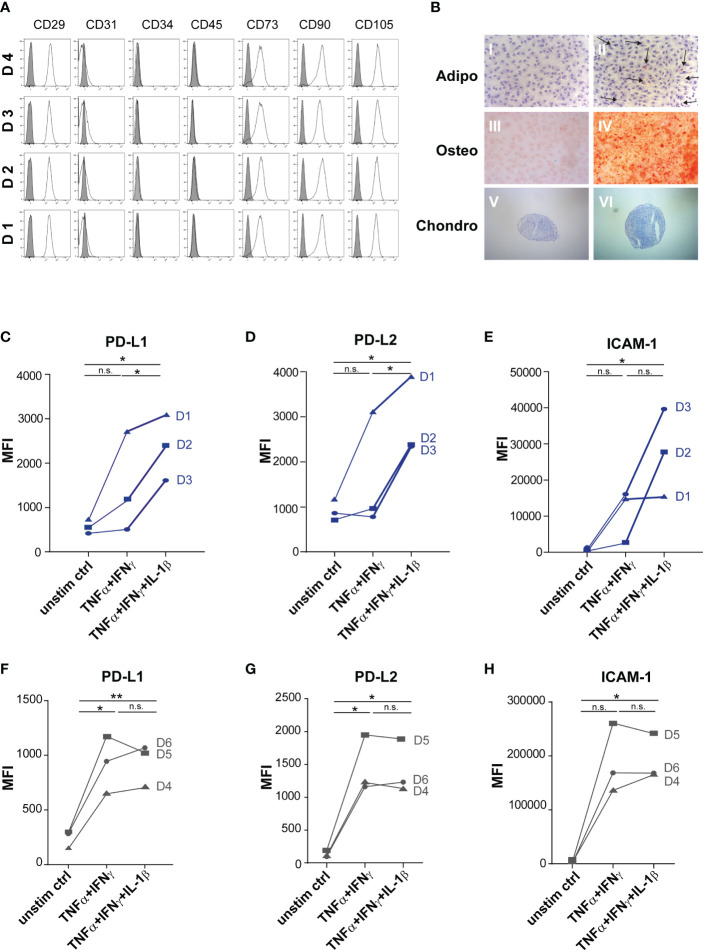
Priming with TNFα, IFNγ and IL-1β significantly increased expression of immuno- modulatory surface proteins of distinct MSCs. **(A+B)** Routine phenotyping after isolation in 2^nd^ passage. **(A)** Flow cytometry analysis of MSCs. Data are shown as an overlay histogram: isotype control (gray) and specific cell-surface markers (white). Cells were labelled with antibodies against CD29, CD31, CD34, CD45, CD73, CD90, and CD105. Dead cells were excluded by live/dead staining. Representative experiments of MSCs used in this study. **(B)** Trilineage differentiation of MSCs. (I+II) Adipogenic differentiation after 14 days of cultivation with standard culture medium or adipogenic induction medium, sudan III staining (arrow: fat vacuoles); nuclei staining with hematoxylin. (III+IV) Osteogenic differentiation after 21 days of cultivation with standard culture medium or osteogenic induction medium, alizarin red staining. (V+VI) Chondrogenic differentiation after 21 days of cultivation in cultivation with standard culture medium or chondrogenic induction medium, alician blue staining. Representative results from MSCs used in the study. **(C–H)** MSCs were stimulated by TNFα (1000 U) and IFNγ (1000 U) or by TNFα (1000 U) and IFNγ (1000 U) in combination with IL-1β (10ng/ml). **(C+F)** PD-L1, **(D+G)** PD-L2, **(E+H)** ICAM-1 expression were measured by flow cytometry, fluorescence mean intensity (MFI): Marker expression minus isotype. **(C-E)** “Full responder” MSCs show further immunoactivating PD-1 ligand expression after triple-priming (D1-D3, in blue) compared to **(F–H)**, (D4-D6). Paired t-test was used to test statistical significance (p < 0.05 considered as significant), data are means (n=3). Data are shown as individual MSC donors. ns, not significant.

The MSCs used in the study were characterized in a standarized procedure and daily routine in our lab according to ISCT criteria (6) (**Figures 1A, B**). Crucial for our study is the immunomodulatory priming of MSCs, which was previously shown to mediate MSCs immunoregulatory activity and specific cell surface markers (45, 46, 53).

In our previous work we observed that during triple cytokine priming by TNF-α, IL-1β and IFN-γ, the cytokine IL-1β further augmented the well-established immunoregulatory activity of MSCs induced by TNF-α/IFN-γ (47). Based on previous studies, we decided to test for PD-l ligand expression of cytokine primed MSCs, as these surface proteins have been described to be crucial for MSCs’ immunoregulatory activity towards T cells (37, 54). We analysed MSCs of different donors with respect to their responsiveness to strong triple-cytokine priming (With 1000 U/ml of TNF-α/IFN-γ and 10ng/ml IL-1β). This response we compared to the well-established dual stimulation with TNF-α and IFN-γ (each 1000 U/ml) (55).

Interestingly, we found two response patterns of MSCs. First, MSCs compiled in **Figures 1C–E** (representative FACS histograms shown in **Figure S1A**) demonstrated a substantial increase in protein expression after triple-cytokine priming compared to the dual priming with TNF-α/IFN-γ alone. These MSCs were considered “full-responders”. In turn, MSCs which were not additionally activated by triple cytokine priming (TNF-α/IFN-γ and IL-1β) and/or showed lower expression of marker proteins in general, were considered as incompletely responsive (**Figures 1F–H**). Further *in vitro* and *in vivo* experiments were conducted with full responder MSC preparations.

**Figure 2:**
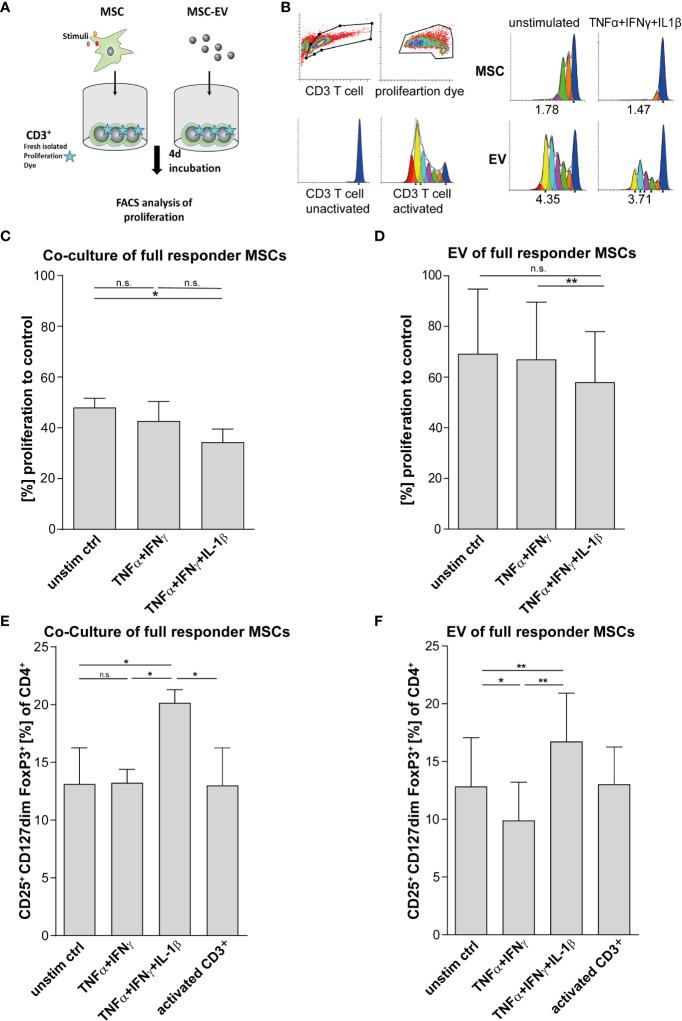
T cell effector function is strongly suppressed *via* direct cell-cell contact and by MSC-EVs after TNFα, IFNγ and IL-1β priming. **(A)** Schematic overview of CD3^+^ proliferation assay. MSCs were primed for 24h by TNFα/IFNγ or by TNFα/IFNγ in combination with IL-1β, cells were washed and cultured in media for additional 48h. For Tcell proliferation assay, CD3^+^ repsonder cells were stimulated with T cell-activating tetrameric antibody-complex CD2/CD3/CD28 and incubated for 4d in the present or absence of different conditioned MSC preparations. **(B)** CD3^+^ proliferation measured *via* dilution of proliferation dye by flow cytometry and proliferation index as indicated below was calculated *via* Modfit software. **(C)** Co-culture of 0.5*10^5^ CD3^+^ and 0.25*10^5^ full responder MSCs (CD3^+^ n = 3), **(D)** 0.5*10^5^ CD3^+^ incubated with EVs isolated from conditioned media of full responder MSCs (MSC-EV n=3; CD3^+^ n=3). Paired t-test was used to test statistical significance (P < 0.05 considered as significant), data are shown as center value: mean; error bars: SD. **(E, F)** For Treg induction assays, MSCs were primed for 24h with TNFα and IFNγ or TNFα, IFNγ and IL-1β SN, cells were washed and additionally incubated in media for additional 48h. Freshly islolated CD3^+^ T cells were incubated for 3d with different MSC preperations. T cells were activated with plate bound antibodies CD3 (1mg/mL) and CD28 (2µg/mL). **(E)** Co-culture of 0.5*10^5^ CD3^+^ T cells and 0.25*10^5^ MSCs selected for subsequent *in vivo* assays (CD3^+^ n = 3). **(F)** 0.5*10^5^ CD3^+^ T cells incubated with EV isolated from conditioned media of full responder MSCs (MSC-EV n = 3; CD3^+^ n = 3). Frequency of Tregs was detected by flow cytometry with MFI marker expression of CD4^+^ CD127^dim^ CD25^+^ FOXP3^+^ of total CD4^+^. Paired t-test was used to test statistical significance (p < 0.05 considered as significant). Data are shown as center value: mean; error bars: SD. ns, not significant.

For validation of MSCs immunoregulatory capacity, we tested the same MSC preparations shown in **Figures 1C–E** and in addition their derived EVs in different immune-functional *in vitro* read-outs based on CD3^+^ T lymphocytes (**Figures 2A–F**). Addition of triple-primed MSC or EVs showed the strongest potential to reduce T cell proliferation (**Figures 2C, D**) and to induce Tregs (**Figures 2E, F**) in CD3 T cell culture assays.

**Figure 3:**
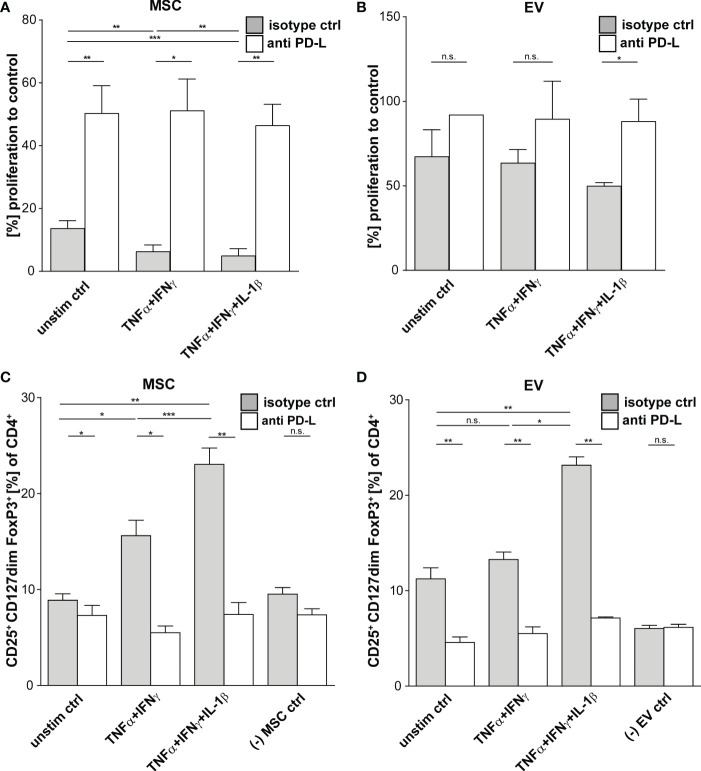
Suppression of CD3^+^ T Cells by primed MSCs/MSC-EVs is mediated by PD-1 Ligands. CD3^+^ isolated from healthy donors were incubated with MSCs/MSC-EVs primed by TNFα and IFNγ or by TNFα and IFNγ in combination with IL-1β and with inhibitory antibodies against PD-L1 (2µg/ml) and PD-L2 (2µg/ml) or isotype control (mIgG1/2a). **(A, B)** CD3^+^ proliferation measured *via* proliferation dye by flow cytometry. CD3^+^ T cells cultured with **(A)** primed MSC or **(B)** MSC-EVs. **(C, D)** Tregs were determined with MFI marker expression of CD4^+^ CD127^dim^ CD25^+^ FOXP3^+^ of total CD4^+^ by flow cytometry. CD3^+^ T cells were cultured with **(C)** MSCs or **(D)** incubated with MSC-EVs. Paired t-test was used to test statistical significance (p < 0.05 considered as significant), MSC, n = 3. Data are shown as center value: mean; error bars: SD. ns, not significant.

Multi-cytokine priming increased PD-L1 and PD-L2 expression in full responder (**Figures 1A–C**). As these proteins have shown strong immunoregulatory activity towards T lymphocytes (34, 35), as proof of concept, we inhibited the function of PD-L1 and PD-L2 on MSCs and EVs by neutralizing antibodies and analysed effects on T lymphocyte proliferation and induction of Tregs. Interestingly, we could showed that PDL blockade restored CD3^+^ T lymphocyte proliferation in co-culture systems with MSC (**Figures 3A, B**). Triple-cytokine stimulated MSCs (**Figure 3C**) and their EVs (**Figure 3D**) strongly augmented induction of Tregs in our *in vitro* system. This induction of Tregs was strongly reduced in the presence of inhibitory antibodies to PD-1 ligands. Interestingly, the Treg induction by unstimulated and dual TNF-α/IFN-γ stimulated MSCs and their EVs was hardly abrogated after PD-L inhibition. Thus, our results support the notion that Treg induction is largely dependent on PD-1/PDL1/2 interaction.

### EVs from immunologically primed MSCs ameliorate murine experimental GvHD

In order to test the therapeutic potential and applicability of the immunologically optimized triple primed MSCs and their EVs, defined by our functional *in vitro* experiments (**Figures 1**–**3**), we utilized a model of experimental murine GvHD. We conducted the following *in vivo* experiments with full responder MSCs after triple-priming compared to unstimulated MSCs and PBS control.

In this model, GvHD is generated by transplanting CD90.2-depleted bone marrow cells (BM cells) from female C57BL/6 donor-mice into female Balb/c recipient-mice, previously irradiated with a total dose of 8 Gy. The recipient female Balb/c mice were reconstituted with BM cells from C57BL/6 mice and with naïve CD4^+^ spleen cells to induce GvHD pathology (51). Groups were treated with MSC-EVs (one injection per day for three consecutive days) or with a single injection of MSCs. Triple-primed MSCs showed an activated phenotype (*in vitro*) and, when injected as whole cells, had a lethal effect on mice directly after intravenous injection, most likely caused by the embolization of lung vessels by this highly activated MSCs.

**Figure 4:**
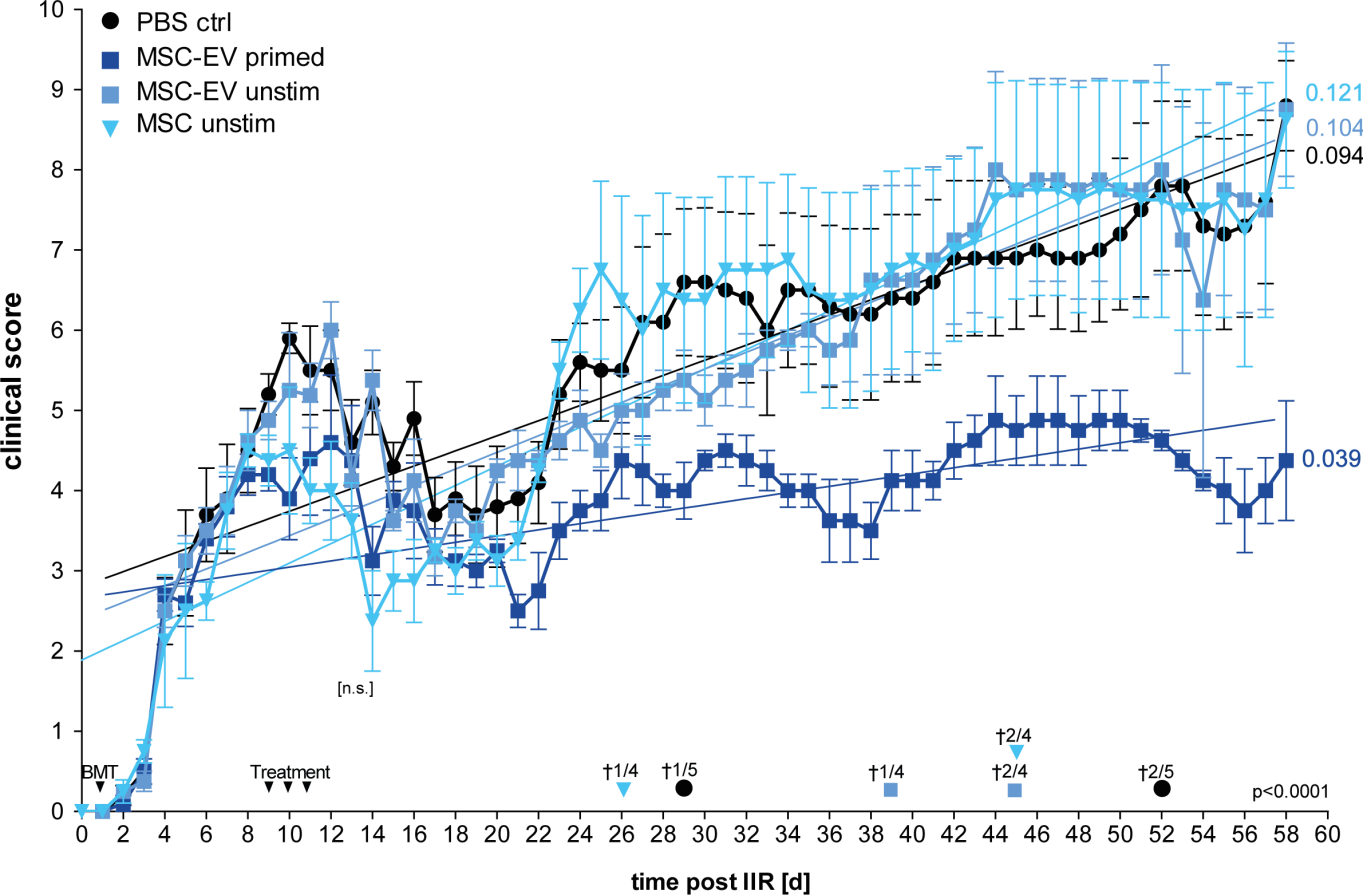
Primed MSC-EVs show long-term beneficial therapeutic effects compared to unprimed MSCs. Balb/c mice were lethally irradiated (day 0) and injected with CD90.2 depleted bone marrow cells and naïve CD4 cells from C57BL/6J mice to induce GvHD (day 1). Treatment with MSC-EVs were performed at day 9, 10 and 11. MSC cells were injected at day 9. Time flowchart of clinical score. Day of deaths and remaining mice per group as indicated. Numbers at the end of linear regressions indicate the slope. P value indicates statistical differences between the groups, [n.s.] on day 13, indicates no significant difference between the groups two days after last treatment, One-way ANOVA with Tukey’s multiple comparison test was used to test statistical significance. Data is shown as center value: mean error bars: SEM.

Thus, in this first set of experiments we compared unstimulated MSCs versus MSC-EVs, and EVs from un-primed versus primed MSCs for the other part (**Figure 4**). From all treatments tested, EVs derived from triple-primed MSCs showed the most beneficial effect in a time course of up to 58 days observation time. At the end of the observation time, we observed a significantly decreased clinical GvHD score, shown with the significant lower slope, after application of triple-primed MSC-EVs compared to all other treatment groups (**Figure 4**).

Additionally, also the overall survival was substantially increased in the group treated with triple primed MSC-EVs (data not shown). Of note, compared to PBS control, the un-primed/resting MSCs (cells) showed a transient decrease in the clinical score directly after treatment (**Figure 4**; at days 8-20). In our priming set-up, only effects of triple-primed MSC-EVs and not primed MSCs (cells) could be analysed as mentioned before.

Next, we considered that PD-L1 and PD-L2 are involved in down-regulating T cell effector function mediating a beneficial therapeutic effect *in vivo*, based on our *in vitro* experiments (compare **Figure 3**). To test this, we treated one group with triple-primed MSC-EVs that were pre-incubated with antibodies directed against PD-L1 and PD-L2 before injection. The second group received primed MSC-EVs pre-incubated with the corresponding isotypes and the control group was treated with PBS + isotype. The unbound antibodies were then removed by 100-kDa molecular weight cut-off (MWCO) centrifugal polyether sulfone membrane ultrafiltration before intravenous injection.

**Figure 5:**
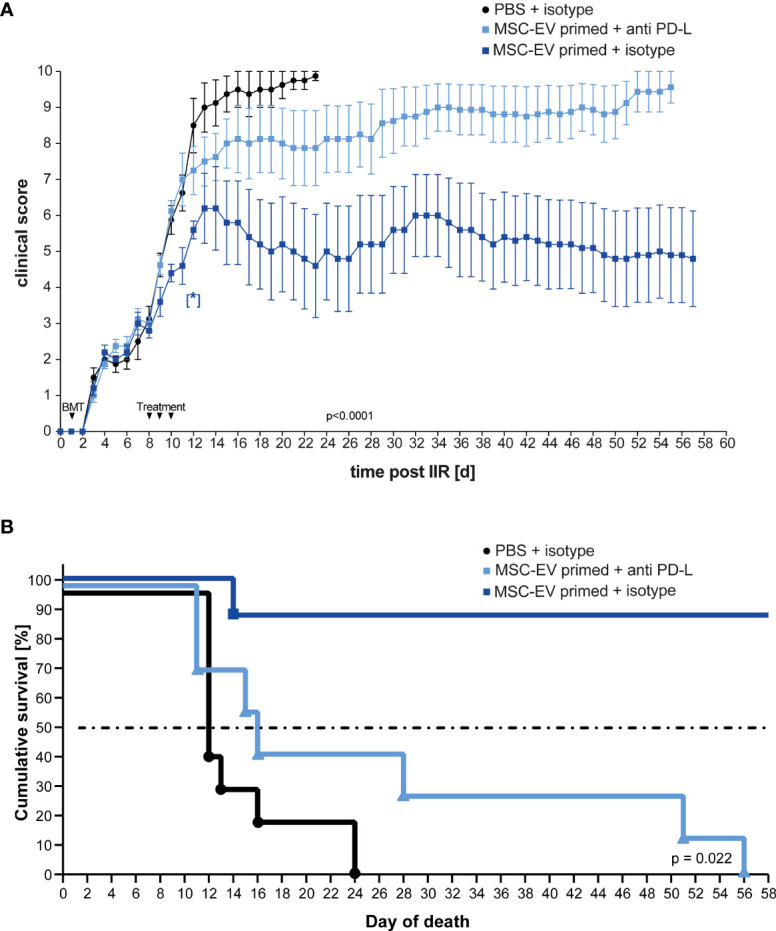
Inhibition of PD-Ligands on primed MSC-EVs abrogates beneficial therapeutic effects. Balb/c mice were lethally irradiated (day 0) and injected with CD90.2 depleted bone marrow cells and naïve CD4 cells from C57BL/6J mice to induce GvHD (day 1) and treated with MSC-EVs of unstimulated MSCs or TNFα, IFNγ and IL-1β stimulated MSCs at day 8, 9 and 10 with primed MSC-EVs pre-incubated with inhibitory PD-1 ligand antibodies or isotype control. **(A)** Time flowchart of clinical score. Data is shown as center value: mean; error bars: SEM. P value indicates statistical differences between the groups until day 24, analysed by linear regression. [*] Indicates p=0,026 significant difference between triple-primed+isotype MSC-EV treated and PBS+isotype treated group two days after last treatment on day 12. **(B)** Kaplan Meier survival curve. n = 8; MSC-EV stimulated + anti-PD-L antibody n = 8; MSC-EV stimulated + isotype control, n = 5. P-value indicates statistical difference for survival between treatment groups, Gehan-Breslow-Wilcoxon test was used to test statistical significance.

In accordance with the previous results, treatment with EVs generated from triple- primed MSCs decreased the clinical score long term (**Figure 5A**). Similar results were obtained when Kaplan-Meier survival analysis was applied (**Figure 5B**). Importantly, the neutralization of PD-1 ligands by blocking antibodies largely abrogated the therapeutic effect (**Figures 5A, B**) suggesting that upregulation of PD-1 ligands substantially contributes to the enhanced therapeutic efficacy of EVs generated from triple-primed MSCs.

**Figure 6:**
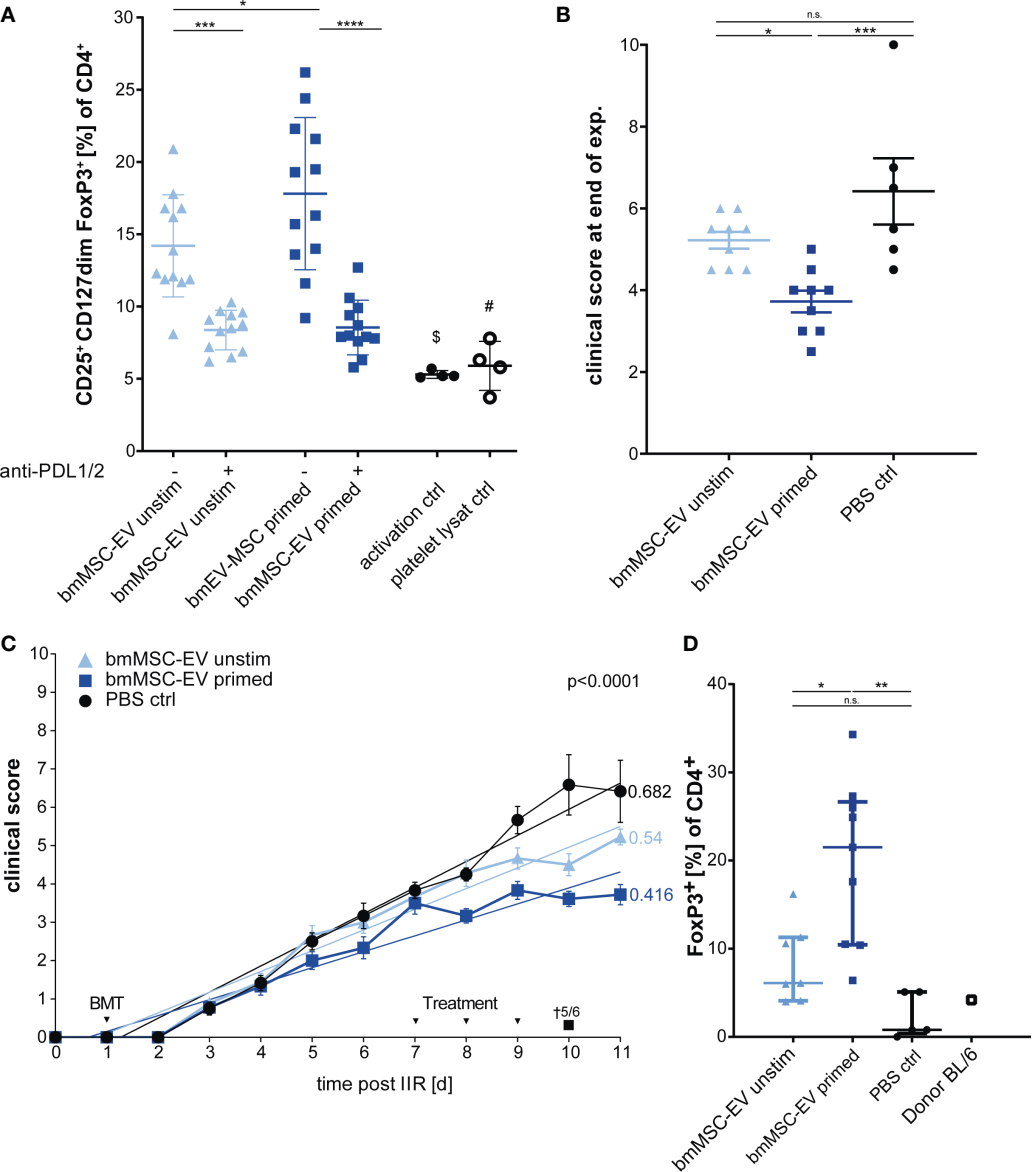
Therapeutic effects can be confirmed with EVs from bone marrow MSCs (bmMSCs). **(A)** Treg induction assay. CD3^+^ T cells isolated from healthy donors were incubated with EVs of unstimulated bmMSCs or TNFα, IFNγ and IL-1β primed bmMSCs. Assay was performed in the presence of inhibitory antibodies against PD-L1 (2µg/ml) and PD-L2 (2µg/ml) or isotype control (mIgG1/2a). Frequency of Tregs were determined by MFI marker expression of CD4^+^ CD127dim CD25^+^ FOXP3^+^ of total CD4^+^ by flow cytometry. CD3^+^ T cells from 4 different donors were tested with 3 different independently cultured and precipitated EV preparations of bmMSC 41.5 batch. Mixed reaction (REML) test was used to test statistical significance, ($) P= n.s., (#) P=0,0248. **(B–D)** Balb/c mice were lethally irradiated (day 0) and injected with CD90.2 depleted bone marrow cells and naïve CD4 cells from C57BL/6J mice to induce GvHD (day 1) and treated with bmMSC-EVs at day 7, 8 and 9. **(B)** Clinical score at day 11 of experiment. One-way ANOVA with Tukey’s multiple comparison test was used to test statistical significance. Data is shown as center value: mean; error bars: SD. **(C)** Time flowchart of clinical score. Data is shown as center value: mean; error bars: SEM. Values on linear regressions indicate the slope. P value indicates statistical differences between the groups analysed by linear regression. **(B, C)** Combined data from two independent animal experiments; bmMSC-EVs unstimulated, n = 9; bmMSC-EV primed, n = 9; PBS control, n = 6. **(D)** Percentage of CD4^+^ FOXP3^+^ Tregs in whole blood after sacrifice. Mice with insufficient blood for further processing are excluded. One-way ANOVA with Tukey’s multiple comparison test was used to test statistical significance. Data is shown as center value: mean; error bars: SD. bmMSC-EVs unstimulated, n = 7; bmMSC-EVs primed, n = 9; PBS control, n = 5. ns, not significant.

In published work, many MSC-based cellular therapies rely on MSCs isolated from bone marrow. Therefore, in a subsequent experiment we aimed to translate our findings based on triple-primed nasal mucosa MSC-EVs to MSC-EVs derived from bone marrow. The bmMSC-EV preparation showed a strong Treg induction *in vitro* which could be significantly enhanced by triple-priming of bmMSCs (**Figure 6A**). Neutralizing antibodies against PD-1 ligands led to significant decrease of Treg induction (**Figure 6A**). In a final series we tested bmMSC-EVs in a short-term GvHD model. Such short-term model enabled us to avoid early death of mice, high clinical scores and offered the possibility to obtain tissue material and peripheral blood from all experimental animals for full comparative analysis between experimental groups.

Using this approach, we found that triple-primed bmMSC-EVs show a similar beneficial therapeutic effect to MSC-EVs from nasal mucosa (**Figures 6B, C**), thus demonstrating that this mechanism is conserved for MSCs isolated from different adult tissue reservoirs. These data demonstrate that immunological priming augments therapeutic efficacy of MSC-EVs from different tissue sources. Interestingly, and despite clear differences of clinical scores in treatment groups, the gut pathology as analysed by swiss roll technology, was not affected by MSC-therapy (**Supplement Figure S3**).

In addition, the short-term model enabled us to analyse the CD4^+^ FoxP3^+^ T lymphocytes in mice blood after scarification at the end of experiment by flow cytometry. The group treated with triple-primed bmMSC-EVs demonstrated the strongest induction of Tregs followed by the unprimed bmMSC-EVs (**Figure 6D**). Tregs might be key cells in maintaining the therapeutic effect in primed bmMSC-EV treated group.

## Discussion

MSC-EVs often recapitulate the immunoregulatory properties of their “parent” cells (20, 26, 27, 38). However, EVs lack the full ability of their parental cells to respond to external signals and thus can only deliver signals and effector molecules already present in their membrane or lumen when generated from their cell of origin (29). Due to the rather short survival time of MSCs in the host (3, 11–13, 23, 24), it is also questionable whether MSCs always receive sufficient priming signals for full immune activation. Against this background, we wanted to develop new priming protocols that robustly enhance the immunoregulatory capacity of MSC-EVs ante partum/prior to therapeutic application. In order to generate “immune enhanced” MSCs, we established a triple-cytokine priming protocol, which enhanced the expression of immunoregulatory proteins associated with MSC’s migration and T lymphocyte suppressor function *via* the PD-1 pathway.

The heterogeneity of MSC therapeutic efficiency, caused by differences in donor and tissue origin as well as isolation and culture procedures, makes it challenging to produce immunoregulatory MSCs with reproducible properties (3, 4, 11–13, 17). Neither searching for surrogate markers to predict MSCs immunoregulatory capacity nor producing immortalized MSCs has led to production of MSC-EVs with robust and reproducible immunoregulatory properties (56). Importantly, within this study we demonstrate that in particular triple-cytokine priming and pre-testing of MSC-EV preparations improve their immunoregulatory properties and can partly overcome MSC heterogeneity and that of their EVs. Nevertheless, as stated and demonstrated by Kordelas et al., the recipient-specific response to primed MSC-EVs has to be elucidated and is of crucial importance (48). Interestingly, we were able to demonstrate that EVs from triple-primed mucosal tissue and bone marrow MSCs significantly increased Treg induction *in vitro* and showed the strongest therapeutic capacity *in vivo*.

Pro-inflammatory stimulation of MSCs has been previously reported to increase PD-1 ligand expression and results in an enhanced suppression of T cell effector function (40, 41, 45, 55, 57, 58). It has also been demonstrated that PD-L1 and PD-L2 function in unison to immune regulate T cells and promote Tregs induction (37, 59). Here, we demonstrate that EVs derived from triple-primed MSCs provide an enhanced clinical outcome in a murine GvHD model, and that this therapeutic effect is at least partly mediated by PD-1 ligands. Our data also support a crucial role of PD-1 ligands on MSCs and MSC-EVs in mediating Treg induction. Of note, TGF-β is abundantly found on EVs and has also shown to immune regulate T cell effector function by inducing Tregs (32). Interestingly, in work related to this study, we found a significant upregulation of TGF-β secretion by MSCs primed by the same multi-cytokine combination used within this study (47).

Stimulation of MSCs lead to enhanced expression of adhesion molecules and to changes in cellular morphology (53, 60). These changes may pose significant risks and side effects especially during intravenous application in the course of cellular therapy (61). In our study, the application of stimulated MSCs also showed a lethal outcome directly after injection in 4 of 5 mice in our GvHD model (data not shown, **Figure 4**), most likely caused by the embolization of lung vessels by highly activated MSCs (11–13, 17). Fatal embolism was described for transfused human decidual stromal cells before in a likely GvHD mouse model (62). These considerations suggest that MSC-EVs may represent a safer and more feasible therapeutic option to prevent therapy-related death (26, 27, 33, 61). Both, in MSC- and MSC-EV-therapy for severe steroid-refractory acute GvHD, the risk for pneumonia-related and mould infection-related death is increased. However, it remains unclear whether these infections owing to the immune-suppressive effect of the steroid therapy, to the immune-regulatory effect of MSCs/MSC-EVs or occurring simply by stochastic risk due to the prolonged survival of patients treated with MSCs/MSC-EVs *per se* (48, 61, 63).

## Conclusion

In this report, we tested dual and triple pro-inflammatory stimulation of MSCs to robustly increase the immunoregulatory properties and in turn to reduce the functional heterogeneity of the parental MSCs and their derived EVs and to study the underlying mechanisms of action in a well-established preclinical GvHD *in vivo* model. Importantly, triple-primed MSCs and their EVs, displayed enhanced therapeutic efficiency, in a PD-1 ligand dependent manner.

## Data availability statement

The original contributions presented in the study are included in the article/**Supplementary Material**. Further inquiries can be directed to the corresponding author.

## Ethics statement

The use of human samples was approved by the ethics committee of the medical faculty of the University Duisburg-Essen. The patients/participants provided their written informed consent to participate in this study. All animal procedures were performed in accordance with the international guidelines for good laboratory practice and the institutional guidelines of the University Hospital Essen approved by the animal welfare committees of North Rhine Westphalia.

## Author contributions

SB, SL, and AH contributed to conception and design of the study. AH, SV, and KB performed the experiments, AH, SV, and SB performed the statistical analysis. AH wrote the first draft of the manuscript. SB and KB wrote sections of the manuscript. All authors contributed to the article and approved the submitted version.
