# Supplementary material for: Immunological priming of mesenchymal stromal/stem cells and their extracellular vesicles augments their therapeutic benefits in experimental graft-versus-host disease via engagement of PD-1 ligands
Source: Front Immunol. 2023 Feb 16;14:1078551. doi: 10.3389/fimmu.2023.1078551 (PMC9978482; doi:10.3389/fimmu.2023.1078551)
Supplement: Supplementary file 1 [file DataSheet_1.pdf]

Supplement

Figure S1. **Flow cytometry gating strategies**. (A) Gating MSC immunoregulatory marker. From left to right: gating in forward scatters for single cells excluding duplets; gating on MSC population by in forward and side scatter; gating on viable cells, excluding cells stained with viability dye 506; Histograms of PD-L1, PD-L2 and ICAM-1 of viable cells. Percentages of total events as indicated. (B) Gating strategy for the detection of Tregs. Representative Tregs gating on lymphocytes of $CD3^+$ Tregs induction assay, shown is medium ctrl after 3d. Lymphocyte population was gated according to forward scatter (FSC) characteristics and side scatter (SSC). Gating on viable cells with viability dye by excluding cells stained with viability dye 506. Gating $CD4^+$ T cells of viable lymphocytes and further separation in $CD25^+$, $CD127^{dim}$, $CD4^+$ and intracellular FoxP3 T Cells. Final gating Treg gating on $CD4^+$ $CD25^+$ $CD127^{dim}$ FoxP3 positive population of $CD4^+$ T cells.

Figure S2. **Analysis of extracellular vesicles of stimulated vs. unstimulated MSCs used in murine GvHD models.** Exemplary analysis of MSC-EVs used in figure 5 and figure 6 is shown. Analysis and characterization were also performed with similar results for all other nmMSC-EVs and bmMSC-EVs fractions presented in this manuscript. (A and D) TEM image of final EV fraction after precipitation, scale bar: 100 nm. (A) Unstimulated MSC-EVs and (D) Stimulated MSC-EVs. (B-E) NTA analysis of final EV fraction after precipitation, particle concentration (particles/ml) and mean diameter (nm) as indicated. (B) EV fraction of unstimulated MSC-EV and (C) EV fraction of stimulated MSC-EVs. (D) Western blot characterization of EV marker proteins in MSCs supernatant (SN) and its corresponding MSC-EVs (EV) fraction for the expression of HSP70, PD-L1, Flotillin-1, CD81 and CD9. Protein concentration was determined with Pierce BCA protein assay kit; 10 µg were applied on SDS gel for western blotting.

Figure S3. Swiss roll analysis of GvHD model

Comparison of colon section of group without GvHD (no GvHD), PBS treated group, unstimulated bmMSC-EV preparations treated or triple cytokine primed bmMSC-EVs preparations treated group, shown in Figure 6 of the manuscript.

Figure S1

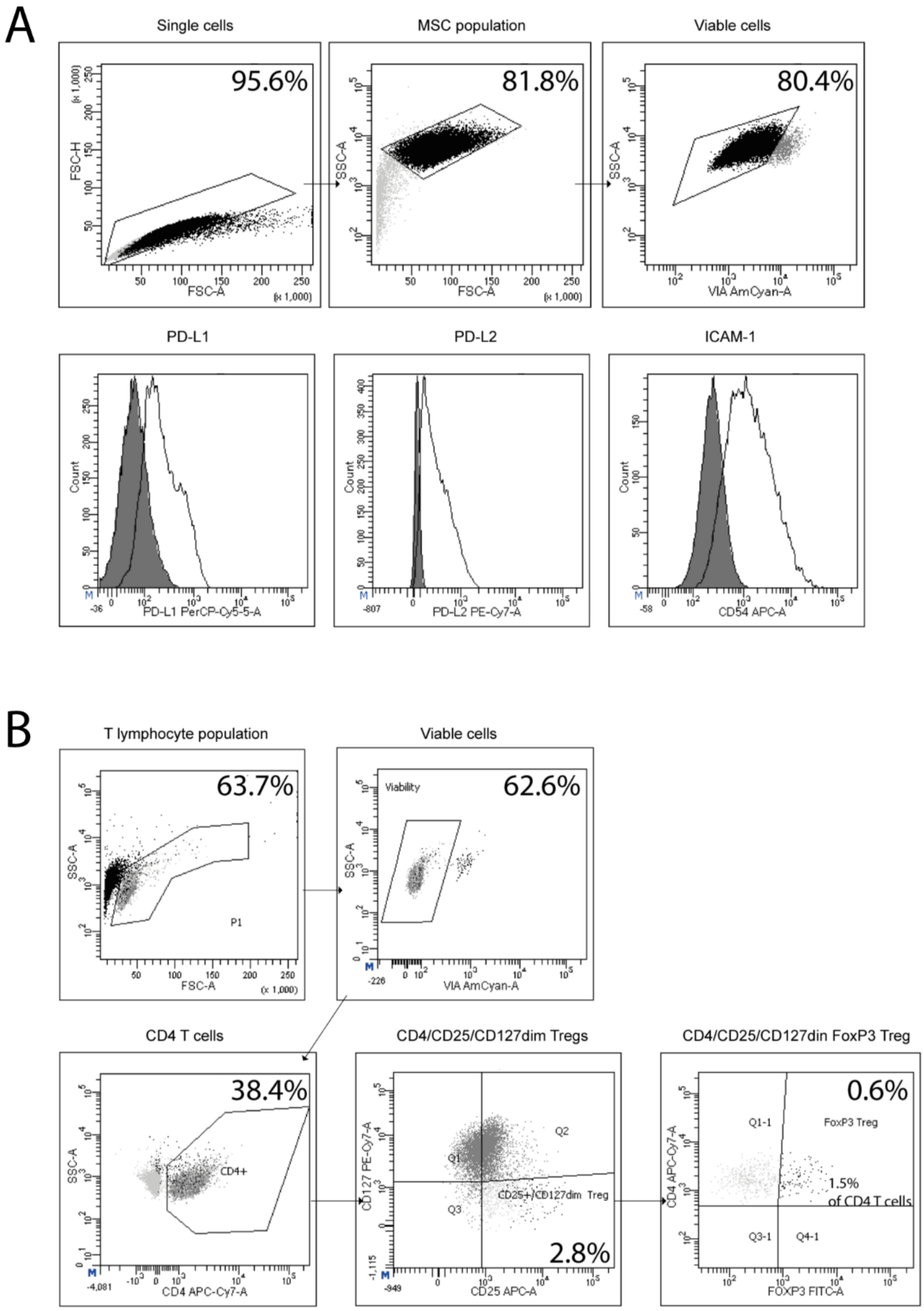

A
Single cells
95.6%
FSC-H
FSC-A
MSC population
81.8%
SSC-A
FSC-A
Viable cells
80.4%
SSC-A
VIA AmCyan-A
PD-L1
Count
PD-L1 PerCP-Cy5-5-A
PD-L2
Count
PD-L2 PE-Cy7-A
ICAM-1
Count
CD54 APC-A
B
T lymphocyte population
63.7%
SSC-A
P1
FSC-A
Viable cells
Viability
62.6%
SSC-A
VIA AmCyan-A
CD4 T cells
38.4%
SSC-A
CD4+
CD4 APC-Cy7-A
CD4/CD25/CD127dim Tregs
Q1
Q2
Q3
CD25+/CD127dim Treg
2.8%
CD127 PE-Cy7-A
CD25 APC-A
CD4/CD25/CD127din FoxP3 Treg
0.6%
Q1-1
FoxP3 Treg
1.5%
of CD4 T cells
Q3-1
Q4-1
CD4 APC-Cy7-A
FOXP3 FITC-A

Figure S2

A

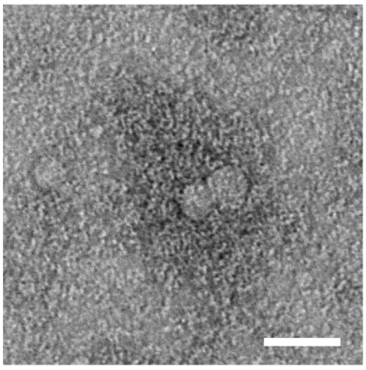

B

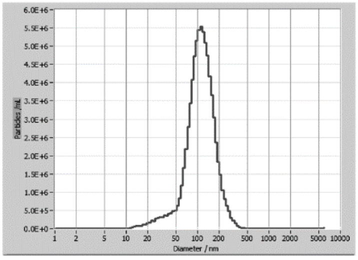

C

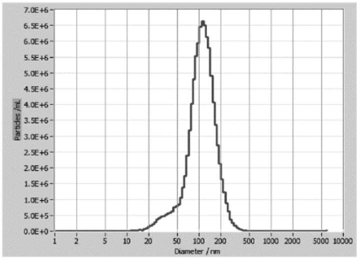

D

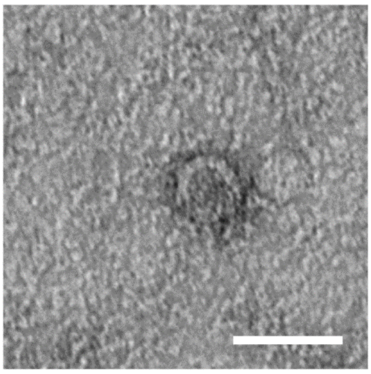

E

Condition	Cytokine-stimulated	Unstimulated
**Particle concentration from NTA**	1.4 x 10^{11} particles/cm^3	1.7 x 10^{11} particles/cm^3
**Average Particle size (X50) from NTA**	107,9 nm	106.3 nm

F

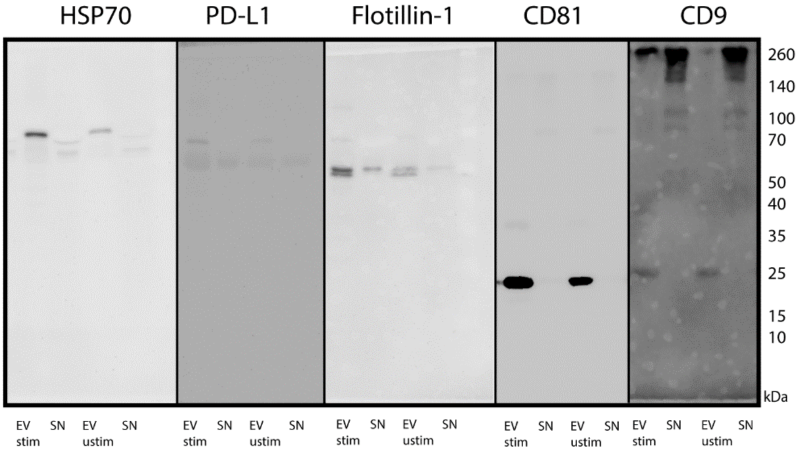

Figure S3

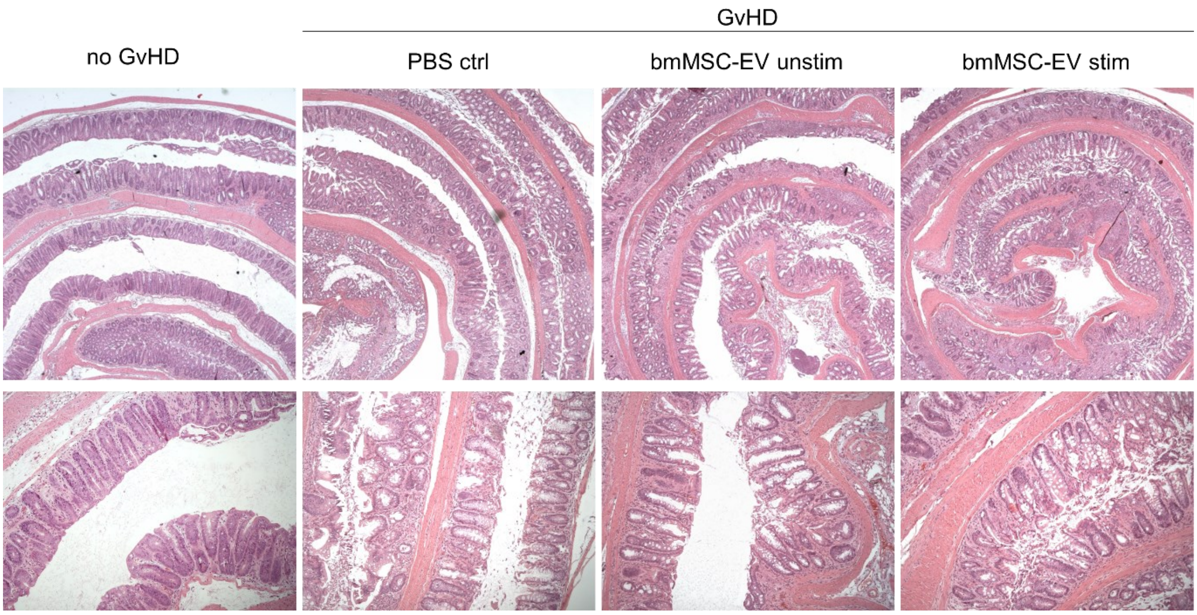

Table S1

Clinical scoring system

Parameter	Clinical Score 0-2 for each parameter<br><br>For dead/sacrificed animals the clinical score was set at 10.	
Weight loss	0	0% - 10%
	0.5	10% - 15%
	1.0	15% - 20%
	1.5	20% - 25%
	2.0	25% - 30%
Fur	0.5	First Indication of less grooming
	1.0	Fussy fur
	1.5	Skin visible on nose and inner femoral fur
	2.0	Total fussy fur and hair loss
Activity	0.5	Less curious
	1.0	Reduced activity

	1.5	No flight reflex
	2.0	Apathetic
Posture	0.5	First indication of hunching
	1.0	Hunching while sitting
	1.5	Hunching while running
	2.0	Barely able to reach for top chow
Skin	0.5	First indication of dandruffs
	1.0	Clear indication of dandruffs
	1.5	Various dandruffs and small scabs
	2.0	Various dandruffs and large scabs

For the clinical scoring the parameters weight, fur, activity, posture and skin were assessed and scored as indicated in the table. Scores for each parameter were added to give the total score (0-10) for an individual mouse. After death/sacrifice a score of “10” was always assigned.
